# Reinforcement Learning in Personalized Medicine: A Comprehensive Review of Treatment Optimization Strategies

**DOI:** 10.7759/cureus.82756

**Published:** 2025-04-21

**Authors:** Banumathi K, Latha Venkatesan, Lizy Sonia Benjamin, Vijayalakshmi K, Nesa Sathya Satchi

**Affiliations:** 1 Community Health Nursing, Apollo College of Nursing, The Tamil Nadu Dr. Maruthur Gopalan Ramachandran (MGR) Medical University, Chennai, IND; 2 Obstetrics and Gynecology, All India Institute of Medical Sciences, New Delhi, New Delhi, IND; 3 Medical Surgical Nursing, King Khalid University, Abha, SAU; 4 Mental Health Nursing, Apollo College of Nursing, The Tamil Nadu Dr. Maruthur Gopalan Ramachandran (MGR) Medical University, Chennai, IND; 5 Child Health Nursing, Apollo College of Nursing, Apollo College of Nursing, The Tamil Nadu Dr. Maruthur Gopalan Ramachandran (MGR) Medical University, Chennai, IND

**Keywords:** artificial intelligence, clinical decision support, digital health, dynamic optimization, personalized medicine, reinforcement learning

## Abstract

Reinforcement learning (RL), a subset of artificial intelligence, is gaining momentum in personalized medicine due to its ability to model dynamic, sequential decision-making. Unlike traditional machine learning approaches, RL systems adapt treatment protocols based on patient-specific responses and evolving health states, offering a robust strategy for optimizing individualized care. This review explores the integration of RL into personalized medicine across diverse clinical domains, including oncology, chronic disease management, psychiatry, infectious diseases, and rehabilitation. Applications such as chemotherapy scheduling, insulin dosing, personalized antidepressant treatment, and ICU management illustrate RL’s capacity to improve therapeutic outcomes by maximizing long-term clinical benefits. Key methodological components, including data integration, reward signal engineering, and interpretability challenges, are discussed alongside solutions such as explainable AI tools, surrogate models, and federated learning. Ethical and regulatory considerations are also examined, highlighting issues such as patient consent, algorithmic bias, and evolving guidelines from regulatory bodies like the Food and Drug Administration and the European Medicines Agency. The review emphasizes the importance of interdisciplinary collaboration and clinician engagement for the successful deployment of RL in healthcare settings. RL presents a transformative framework for delivering adaptive, equitable, and patient-centered treatment strategies. Future research should focus on implementing it safely, scalably, and transparently to fully harness its potential.

## Introduction and background

In the world of medicine, personalized medicine, also known as precision medicine, represents a paradigm shift that focuses on providing tailored medical treatment based on a patient’s unique characteristics. In contrast to conventional medicine, which employs a one-size-fits-all approach, personalized medicine utilizes a patient's unique characteristics, such as genetics, molecules, environment, and lifestyle, to identify the key factors that inform an accurate diagnosis, prognosis, and treatment selection. In oncology, cardiology, endocrinology, and neurology, areas where heterogeneity in disease expression and response to treatment is particularly large, this approach has proven extremely useful [1-3].

Advances in biomedical research and the accessibility of high-throughput data contributed to the application of precision medicine in clinical practice. However, the patient data is massive and complicated to analyze; it requires the use of complex computational tools to extract useful insights and make actionable recommendations [2].

The analysis of healthcare data using state-of-the-art technology, such as artificial intelligence (AI) and machine learning (ML), is the payoff. AI systems are currently being used routinely for disease detection, radiological imaging, genomics, and treatment planning, offering unprecedented diagnostic accuracy and efficiency [3-6]. The fact that the AI-driven methods can, in many cases, identify patterns in large and complicated datasets makes them a very suitable tool for applications where we want to integrate and interpret multidimensional data in personalized medicine.

Reinforcement learning (RL) is becoming dominant due to its capacity to learn optimal strategies in dynamic and uncertain environments. RL models improved decision-making over time by taking feedback from the environment and learning interactively [4-6]. The fact that RL is well-suited to clinical situations where treatment decisions need to adjust to patient responses and disease progression marks a strong advantage in applying RL to optimize health outcomes.

RL is a type of ML motivated by behavioral psychology. It is an agent that interacts with the environment to maximize the cumulative reward by taking actions. The agent, environment, state, action, reward, and a policy mapping states to actions are key components. The agent is a decision-making algorithm, and the environment can include patient characteristics and disease states in medical applications. For example, the reward function typically corresponds to a clinical outcome, such as symptom relief, survival, or normalization of a biomarker [5,7-9].

Q-learning is an example of a value-based method, policy gradients are an example of a policy-based method, and actor-critic models are an example of a hybrid method [10]. Deep RL (DRL), which combines deep neural networks with the principles of RL, has been influential in handling complex and high-dimensional healthcare data [6]. These can capture patient dynamics and produce not only personalized treatment strategies but also adapt as their understanding of the patient evolves.

The objective is to gain a thorough understanding of the principles of RL and explore its potential application in personalized clinical treatments within clinical environments. Specifically, this involves exploring the use of RL in multiple medical fields, such as oncology, chronic disease management, and psychiatry, and detailing relevant case studies and real-world examples in these areas where it is applied. Furthermore, upon integrating RL into personalized medicine, the issues and areas of interest that arise from this integration will also be discussed, along with future research directions.

## Review

Methods

RL in personalized medicine has been extensively used over the past few years, during which this narrative review was conducted, to present a comprehensive synthesis of recent applications of RL in personalized medicine. Although no systematic review protocol was followed, a structured approach was used to identify, evaluate, and select relevant literature.

Literature search strategy

A thorough literature search was conducted in the aforementioned databases, including PubMed, Scopus, IEEE Xplore, and Google Scholar, for relevant studies published between January 2015 and March 2025. Keywords and Boolean operators (“reinforcement learning” OR “Q-learning” OR “deep RL” OR “actor-critic” OR “policy gradient”) AND (“personalized medicine” OR “precision medicine” OR “clinical decision support” OR “adaptive treatment” OR “healthcare”) were utilized in the search strategy. The reference lists of key reviews and high-impact publications were manually screened for additional articles. In the survey, peer-reviewed journal articles, proof-of-concept trials, and clinical studies that used RL in real-world or simulated medical settings were given priority. In contrast, papers that used RL on a simulation were given lower priority and were ultimately excluded from the analysis.

Inclusion and exclusion criteria

Studies included in this review focus on the applications of RL in personal healthcare, including oncology, diabetes management, psychiatry, and critical care, among others. Eligible articles also explain specific RL algorithms, including Q-learning, deep Q-networks, policy gradient techniques, actor-critic architectures, or hybrid models. Additionally, studies utilizing clinical data, clinically relevant simulations, or RL integration in decision support systems were considered. Exclusion criteria involved studies that focus solely on supervised or unsupervised ML without any RL components. Articles not available in English, as well as non-peer-reviewed commentaries, editorials, and whitepapers, were also excluded from this review. For study selection and organization, methodologically clear and relevant full texts were reviewed. Medical domains and RL techniques were grouped thematically to highlight the breadth of application contexts and the diversity of RL methodologies employed.

Fundamentals of reinforcement learning

Core Concepts of Reinforcement Learning

RL is a goal-directed learning framework in which an agent interacts with the environment and learns the optimal actions based on the rewards it receives. An RL framework consists of an agent, an environment, a state, an action, and a reward. The agent views the current state of the environment, chooses an action, and then receives a reward, transitioning to a new state as a result of that action. Over time, an agent learns the policy, a strategy that maps states to corresponding actions, which maximizes the cumulative reward [6]. The agent can be a decision support algorithm in a clinical context, and the environment comprises patient data and health status. Patient vitals, lab results, or disease stage may be included as states, and medication adjustments, procedural interventions, or diagnostic tests may be actions. The reward is usually a surrogate for clinical outcomes, such as symptom improvement, survival, or disease remission [7]. Indeed, this structure aligns well with dynamic and personalized medical decision-making, particularly when treatments are adjusted repeatedly based on the patient’s condition.

Types of Reinforcement Learning

Model-free and model-based are the two types of RL algorithms [9]. Model-free methods do not require knowledge of the environment's transition dynamics; instead, they learn optimal policies from experience. On the other hand, model-based RL uses a simulation (system dynamics or event-driven) model of the environment to simulate outcomes, which can help improve learning efficiency. However, modeling biological systems is very complex in medical applications [8]. Value-based, policy-based, and actor-critic methods are further classifications of RL strategies. Q-learning and SARSA are value-based methods that aim to learn a value function that estimates the expected reward of taking an action in a given state [10]. Since the value of actions can be explicitly defined in problems like treatment scheduling and medication dosing [9], these methods are instrumental in such contexts. Lastly, we look at policy-based methods, which do not require value estimation and directly optimize the policy. It is helpful for high-dimensional or continuous action spaces, which appear, for instance, in robotic surgery or personalized medication dosing [10]. Actor-critic methods combine value- and policy-based approaches by using a value function (critic) and a policy (actor), leading to more stable learning and promising potential in healthcare applications [11]. Inspired by the latest technological advancements in DRL, which involve integrating deep neural networks into RL techniques, the field has taken a new path toward automation. Due to its ability to handle high-dimensional inputs, such as medical images, electronic healthcare records (EHRs), and multimodal clinical data, DRL is very effective in tasks like optimizing radiation treatment protocols, predicting patient trajectories in critical care, and designing adaptive treatment policies in chronic diseases [12]. Recently conducted proof-of-concept trials have demonstrated the real-world clinical utility of RL in providing personalized treatment, especially in cases such as adaptive androgen deprivation therapy for prostate cancer and glycemic control strategies for type 2 diabetes, where RL-based optimization has been effective [13,14].

Comparison With Other Machine Learning Paradigms

Traditional ML approaches, including supervised and unsupervised learning, have achieved significant success in disease classification, imaging diagnostics, and risk stratification. However, they cannot perform real-time treatment optimization because they cannot make sequential decisions [15]. Since the operating point changes over time, and the model therefore does not have access to a static dataset, there is no fixed mapping from input to output. Thus, supervised models cannot adapt to changes in patient condition or treatment effects. On the other hand, RL provides a dynamic environment for learning optimal actions through continuous interaction with the environment. In personalized medicine, the efficacy of treatment varies significantly among individuals, and timely intervention becomes necessary to respond to changing clinical contexts [16]. This adaptability is crucial. A comparison of RL with other commonly used ML methods in healthcare is presented in Table 1, outlining key attributes in terms of functionality, learning methodology, and clinical applicability.

**Table 1:** Comparative overview of ML paradigms in healthcare ML: machine learning, RL: reinforcement learning, ICU: intensive care unit

Learning paradigm	Key features	Example clinical application	Limitations in healthcare context	Reference
Supervised learning	Learns from labeled datasets	Disease classification (e.g., cancer detection)	Cannot adapt to dynamic patient responses	Jayaraman et al., 2024 [6]
Unsupervised learning	Finds hidden patterns in unlabeled data	Patient subgrouping or clustering	No direct decision-making capabilities	Daidone et al., 2024 [15]
RL	Learns via trial-and-error feedback	Adaptive therapy planning	Requires careful reward design	Liu et al., 2022 [7]
Deep learning	Extracts feature from high-dimensional data	Radiology and pathology image analysis	Opaque decision-making ("black box")	Lu et al., 2024 [12]
Semi-supervised learning	Combines small labeled with large unlabeled datasets	Risk prediction with limited labels	Sensitive to noisy, unlabeled data	Huang et al., 2022 [14]
Transfer learning	Leverages knowledge from other domains	Adapting models between populations	May transfer biases inadvertently	Wang et al., 2023 [13]
Online learning	Updates in real-time with new data	Wearable health monitoring systems	Prone to concept drift without oversight	Liu et al., 2020 [11]
Model-based RL	Learns the environment model before acting	ICU policy simulation	Requires accurate patient modeling	Limonte et al., 2022 [8]
Model-free RL	Learns directly from experiences	Glucose regulation in diabetes	Slow convergence in sparse reward cases	Loftus et al., 2020 [10]
Hybrid models	Combines multiple ML paradigms	Personalized drug recommendations	Complex training and interpretability	Jafar et al., 2024 [16]

Personalized medicine: an overview

Definition and Evolution of Personalized Medicine

Personalized medicine, also known as precision medicine, is a new and transformative way of delivering healthcare, where decisions regarding medical interventions and healthcare practices are made on a person-by-person basis. While conventional "one-size-fits-all" medicine does not account for differences in people’s genetic makeup, environment, and behavior, personalized medicine does. However, significant advances have been made over the past decade due to developments in genomics, bioinformatics, and systems biology [17]. In oncology, there has been a significant surge in this impact, with the genetic profiling of tumors allowing oncologists to choose therapies based on specific molecular targets. Pharmacogenomics used in neurology is helpful to foretell which group of patients will respond best to antidepressants or antiepileptics. In general, integrating patient-specific biological data enables clinicians to create precise, patient-centric therapies that offer maximum benefit and minimal harm [18].

Key Components and Goals

The three areas in which the extension of personalized medicine is genomic medicine, predictive modeling, and tailored therapeutics. With the mapping of the human genome and advancements in next-generation sequencing technologies, genomic medicine has made it possible to identify genetic variations that influence susceptibility to and responses to various diseases and treatments. This is particularly important in oncology, cancer treatment, hereditary diseases, and predicting rates of drug metabolism [19], where genomic information is used to select targeted treatments. Predictive modeling is a form of data analytics or ML that creates models to predict the onset, progression, and potential outcomes of treatments or future disease occurrences. Due to these reasons, these models are used extensively in early diagnosis, risk stratification, patient monitoring, and prevention strategies [20]. The field of personalized therapeutics is much broader than genetic information and also encompasses clinical biomarkers, physiological data, and real-world patient behavior. By doing so, this approach guarantees that interventions are tailored to the individual patient’s needs in terms of intensity and timing [21].

Data Sources in Personalized Medicine

For personalized medicine purposes, data plays an indispensable role, allowing for the use of numerous data sources, ranging from biological and clinical data in the case of algorithms to clinical treatment decisions, which facilitate this. EHRs are longitudinal records of patient data, including demographics, clinical encounters, medications, lab results, imaging reports, and other health IT applications such as care plans, telemedicine, telehealth, clinical decision support, and e-prescribing. However, these datasets are useful for monitoring patient progress over time and gauging patient reaction to treatment [22]. These processes utilize high-throughput platforms that can generate large amounts of data related to genomics, proteomics, metabolomics, and transcriptomics, aiding in the discovery of disease biomarkers and therapeutic targets. The roles of these "omics" profiles are indispensable for personalized treatment [23]. Furthermore, wearables and remote monitoring devices, such as smartwatches and fitness trackers, can offer continuous monitoring, providing real-time data on physiological factors (e.g., heart rate, glucose levels) and physical activity metrics. The data streams enable the collection of patient adherence data, recovery trend data, and early signs of deterioration data, providing a further mechanism for offering individualized care [24].

Challenges in Current Personalized Treatment Planning

Personalized medicine has several hurdles. First of all, the existence of data silos and issues of interoperability impede the seamless exchange and integration of different sets of data. Second, data quality and completeness are often incomplete or inaccurate, especially in EHRs, and reasonable uncertainty may exist regarding model accuracy [24]. In addition, interpretability and clinical actionability are still problems. For recommendations from ML models to be accepted, clinicians must understand and trust them. As such, the models must be transparent and explainable. Secondly, we discuss ethical issues, such as data privacy, informed consent for genomic testing, and the risk of algorithmic bias [25,26]. Lastly, economic and infrastructural constraints limit the scalability of personalized medicine at the final stage. Genomic testing and advanced analytics are expensive and only accessible in urban centers or academic hospitals.

Why Reinforcement Learning for Personalized Medicine?

RL is applied to personalized medicine because personalized treatment is a sequential decision problem. Caring for patients is a natural sequential decision-making problem in which treatments are changed over time, and each clinical decision influences the future patient states. An example of this is in chronic disease management, oncology, and critical care, where treatment regimens, such as dosage, combination, and timing of medication, often need to be modified to ensure the best response in a patient at a given point in time [27]. However, traditional statistical models are not sensitive to capturing such evolving dynamics over time. In particular, RL is well suited to solve personalized, sequential decision-making problems [28].

In the healthcare environment, there is high uncertainty and variability in how patients respond to treatments, particularly in dynamic and uncertain settings. Patients faced complications in treatment outcomes due to unexpected events and delayed adverse effects. Because of this uncertainty, RL can adapt to changing conditions by continually updating its understanding of the environment. Also, RL algorithms have been applied to determine the optimal timing of starting or stopping ventilator support in a critical care setting and estimate insulin doses based on physiological feedback [29]. In sepsis management, for example, or in corticosteroid therapy [30], RL has been shown to outperform physician-driven policies by identifying adaptive treatments based on observed outcomes. In particular, the flexibility with which RL can handle partial information and delayed feedback is well-suited for clinical scenarios and can be leveraged to develop robust decision support tools that have to adapt as the patient situation evolves.

Patient variability is a significant factor in personalized medicine, and RL also takes it into account. Patients' genetic backgrounds, disease courses, lifestyles, and treatment compliance are different. The RL algorithm can handle this heterogeneity for learning patient- or subgroup-specific policies. Unlike traditional approaches, such as maximizing the average outcome, RL seeks to maximize individual rewards, thus following clinical endpoints like symptom reduction, improved quality of life, or survival [31]. For instance, RL is used to personalize insulin dosing for patients with type 1 and type 2 diabetes, as well as dietary decisions. It has also been shown recently that RL can adjust insulin doses in real-time, depending on meal composition, activity, and glucose levels, which significantly improves glycemic control [32,33].

Furthermore, RL models have been demonstrated to be able to manage multimorbid conditions by considering the drug interactions and comorbidities [34]. RL has been used in cancer treatment to recommend intermittent androgen deprivation therapy for prostate cancer to achieve the best balance between tumor control and side effects [35]. These diseases are long-term in nature, and treatments are cumulative; therefore, RL’s long-term optimization perspective is critical.

Let’s now compare RL with other types of learning, such as supervised and unsupervised ones, and see what nice gains we obtain with RL. However, supervised learning has been helpful in diagnostic classification and risk prediction. However, relying on static labeled datasets and being unable to model interventional outcomes has minimized the efficiency of the designed methods. Unsupervised learning is helpful for clustering or identifying hidden patterns in data, but it does not directly impact decisions. Whereas, RL is interventional and adaptive by its essence. It learns from observed data and real-time interactions and recommends and evaluates the consequences of specific clinical actions [36]. This capability makes RL an alternative technology that resides between predictive analytics and action recommendations. Moreover, it offers a dynamic layer for AI-powered personalized medicine by incorporating temporal dependence and decision feedback, which are usually ignored by conventional ML techniques [37,38].

Applications of Reinforcement Learning in Personalized Treatment Protocols

**Figure 1:**
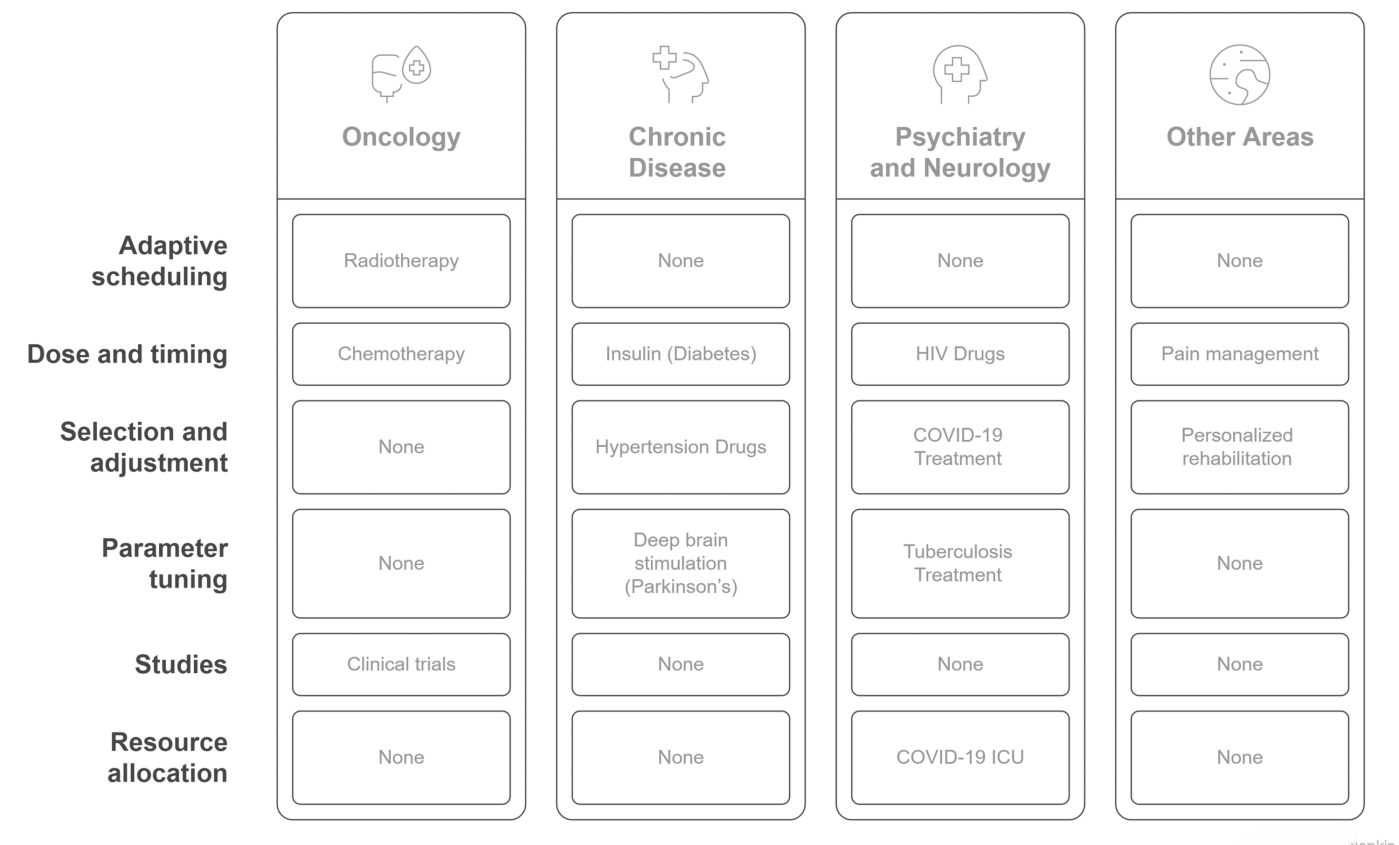
Applications of RL in personalized treatment protocols Image Credit: Banumathi K (Author) RL: reinforcement learning, COVID-19: coronavirus disease 2019, ICU: intensive care unit

Figure 1 illustrates the diverse clinical applications of RL in personalized medicine, showcasing its implementation across oncology, chronic diseases, psychiatry, infectious diseases, and rehabilitation settings.

Oncology: In oncology, treatment personalization is essential due to the range of possible biological tumor types, a patient's genetics, and their response to therapy. Adaptive radiotherapy scheduling is achieved through algorithms in RL that plan the timing and doses of radiation for treatment based on its relationship to tumor growth models, tissue tolerance, and personal response patterns. RL uses optimal policies that adjust based on tumor shrinkage and toxicity data to balance safety and efficacy [37]. Conventional chemotherapy is delivered on a rigid schedule, and there may be over- or under-treatment. RL-based frameworks, such as Q-learning and deep Q-networks, have led to practical case studies and clinical trials for simulating the implementation of chemotherapy protocols and adaptive hormone therapies for prostate and breast cancers. The medical community continues to evaluate these systems for their potential use in actual oncologic decision processes [39].

Chronic disease: RL is beneficial for learning, as treatment regimens for chronic diseases need to be adapted over time. In particular, real-time insulin dosing for patients with type 1 and type 2 diabetes is a notable application. RL models sample food intake, glucose patterns, and movement-based activities to determine the optimal amount of insulin to inject. Effective glycemic control has been demonstrated in real-world trials using insulin pumps driven by RL or decision support systems [40]. Likewise, in hypertension, where personalized drug selection and continuous dosage adjustment are necessary, RL models have developed optimal treatment policies for lowering blood pressure while minimizing side effects in patients with diabetes and renal impairment [41]. For example, RL models are being trained to personalize how individuals use inhaled corticosteroids based on symptoms, spirometry data, and environmental triggers, aiming to improve patient compliance and reduce medication titration and adherence exacerbations for chronic obstructive pulmonary disease and asthma [42].

Psychiatry and neurology: These psychiatric and neurological disorders are highly heterogeneous and exhibit a slow response to treatment, making them excellent candidates for RL-based approaches. Traditionally, antidepressant medication personalization utilizes trial-and-error methods for prescribing antidepressants. RL can optimize regimen choices by integrating patient history, genetic markers, and longitudinal mood data to predict the best treatment options for each individual [43]. In epilepsy, RL is applied to personalize the setting of anti-seizure medication or neurostimulation based on predictive models of seizure likelihood, enabling intervention before seizures occur and thus reducing seizure frequency [44]. Additionally, RL extends to deep brain stimulation (DBS) parameter tuning, which involves adjusting stimulation parameters to achieve maximum motor control with minimal side effects, including dyskinesia and cognitive dysfunction, in DBS patients [44].

Infectious disease: Treatment responses are variable, and resistance patterns of infectious agents evolve, making RL highly applicable. Specifically, in optimizing antiretroviral therapy [42], RL models develop treatment plans based on patient adherence, viral load dynamics, and the potential for novel mutations leading to drug resistance, aiming to improve long-term viral suppression. RL has been used during pandemics to guide patient-specific ICU interventions, such as selecting ventilation strategies and medications based on dynamic information, including oxygen levels and inflammation markers [39]. Furthermore, RL strategies have shown benefits in tuberculosis treatment [45], allowing for the adjustment of treatment regimens to individual pharmacokinetics and adherence patterns, which can improve cure rates in patients with multidrug-resistant tuberculosis. Table 2 illustrates how RL operates in medical settings by comparing different domains based on their decision-making approaches, model types, and achieved outcomes.

**Table 2:** Applications of RL in clinical domains RL: reinforcement learning, DRL: deep reinforcement learning, COPD: chronic obstructive pulmonary disease, COVID-19: coronavirus disease 2019, ICU: intensive care unit

Domain	Use case example	RL technique applied	Clinical benefit	Reference
Oncology	Adaptive radiotherapy	Deep Q-learning	Dose optimization with minimized toxicity	de Giorgi et al., 2022 [37]
Oncology	Chemotherapy scheduling	Model-based RL	Reduced side effects, improved efficacy	Anzabi et al., 2023 [38]
Oncology	Prostate cancer hormone therapy	DRL	Adaptive hormone suppression	Zheng et al., 2021 [39]
Diabetes management	Insulin pump regulation	DRL	Stable glucose levels	Liu et al., 2024 [40]
Hypertension	Personalized drug titration	Actor-critic models	Controlled blood pressure	Badjatia et al., 2025 [41]
Asthma/COPD	Inhaler adherence optimization	Policy gradient methods	Reduced exacerbations	Munson et al., 2024 [42]
Depression	Antidepressant sequencing	Multi-arm bandit RL	Enhanced treatment response	Sun et al., 2022 [43]
Epilepsy	Seizure intervention timing	Q-learning	Lowered seizure frequency	Kourou et al., 2021 [44]
Parkinson’s	DBS parameter optimization	DRL	Improved motor function	Kourou et al., 2021 [44]
COVID-19 ICU	Dynamic treatment planning	Model-free RL	Resource-efficient ICU management	Zheng et al., 2021 [39]

Data requirements and integration

Types of Data Needed for Reinforcement Learning Models

In personalized medicine, RL is very effective when the quality, diversity, and continuity of patient data are sufficient. RL models that require proper capture of clinical state complexity and treatment outcome complexity require datasets that explicitly enable those. RL inputs are composed of structured and unstructured data. Such structured data includes laboratory values, vital signs, and a medication list, all of which are vital for systematic decision-making. Yet, structured formats often miss the wealth of context in unstructured data, such as clinical notes, radiology reports, and patient stories. Natural language processing (NLP) techniques are applied to the RL system to interpret and utilize unstructured data for state and reward modeling, which improves the overall effectiveness of RL models in personalized medicine [31]. Another significant component is the continuous and real-time data streams, which enable the transmission of real-time data from continuous glucose monitoring, electrocardiogram signals, and wearable devices, facilitating real-time updates to treatments using RL algorithms. As these are continuous data streams, they offer opportunities for fine-grained learning and decision-making in critical care, diabetes management, and rehabilitation scenarios [32].

Data Preprocessing and Standardization

Before feeding raw clinical data into RL models, the data is often incomplete, noisy, and inconsistent and requires preprocessing. A common problem in healthcare is handling missing data and noise, which can cause bias in learning processes. Techniques such as preprocessing, including imputation and noise filtering, can be used to address these challenges. Moreover, time series alignment is also necessary for synchronizing asynchronous clinical events for coherent state transitions [33]. Furthermore, RL state feature extraction is crucial in identifying the features relevant to patient states. The key to feature engineering is deriving composite variables that capture meaningful physiological or pathological conditions. Additionally, using convolutional or recurrent neural networks in DRL setups, the model can learn the dimensions of automatically extracted features from raw data, including images or waveforms. However, this process allows the model to utilize all the information in the raw data, rather than a small set of manually selected features, as is the case in supervised learning [34].

Real-Time Feedback and Reward Signal Engineering

RL is a hallmark of being able to evaluate actions concerning reward signals. Since clinical outcomes must be meaningful and aligned with the treatment goals in healthcare, designing a reward function is crucial. In medical RL systems, the design of reward functions can be related to short-term goals, such as reducing pain scores or glucose levels, or long-term outcomes, such as survival or disease remission. Many tradeoffs must be balanced in the reward structure, whereby the reward function should maximize treatment efficacy while minimizing side effects [35]. However, high reward delay and sparsity present significant challenges in clinical RL. For example, in the treatment of chronic diseases or cancer, the effects of treatment may take weeks or even months to show, and thus convergence may be slow, or policies may be close to suboptimal if rewards are sparse. To overcome these challenges and improve the learning process in such long-term treatments, techniques such as reward shaping and using intermediate proxy outcomes (e.g., changes in biomarkers) can be used [36].

The primary data types used in RL applications in healthcare are summarized in Table 3, along with the challenges they present for processing and integration.

**Table 3:** Data types and requirements for RL in personalized medicine RL: reinforcement learning, EMRs: electronic healthcare records, PET: positron emission tomography, ICU: intensive care unit, NLP: natural language processing, CNNs: convolutional neural networks

Data type	Clinical source	Key use in RL	Integration challenge	Reference
EMRs	Hospital EMRs	Define state-action transitions	Inconsistent formatting, missing data	Engelhardt and Michor, 2021 [31]
Genomic data	Whole genome sequencing	Patient stratification	High dimensionality	Mushtaq et al., 2023 [23]
Imaging data	Radiology/PET scans	Deep RL model input	Requires CNNs for interpretation	Lu et al., 2024 [12]
Time-series vitals	ICU monitors	Reward function adjustment	High frequency, noise interference	Zhang et al., 2025 [32]
Lab results	Diagnostic labs	Outcome assessment and feedback	Standardization across labs	Mushtaq et al., 2023 [33]
Medication records	Pharmacy data	Treatment history tracking	Polypharmacy complexity	Cheng et al., 2021 [25]
Behavioral data	Wearables, activity trackers	Real-time context awareness	Data overload and user variability	Grolleau et al., 2024 [24]
Physician notes	Clinical documentation (NLP)	Contextual insight for decisions	NLP is required for unstructured text	Engelhardt and Michor, 2021 [31]
Environmental data	Geolocation and pollution monitors	Risk factor modeling	Sparse or indirect impact on treatment	Coronato et al., 2020 [26]
Biomarker panels	Proteomics and metabolomics	Disease state classification	Feature extraction challenges	Giordano et al., 2021 [34]

Model interpretability and clinical integration

Black-Box Challenge in Deep Reinforcement Learning

DRL models have achieved excellent results; however, they very often suffer from the so-called black box problem, as they are not transparent. The lack of transparency can be a significant barrier in the clinical world; the stakes are high for mortality, as in cancer treatment or critical care. This is why clinicians are naturally reluctant to trust and use models they cannot fully understand [39]. In addition, DRL models are difficult to explain due to the complex relationships between neural network parameters and the policies they learn. With no dedicated interpretability mechanisms, it is challenging to understand why a specific action was taken in a particular state and thus how clinicians can justify the decisions made by these models.

Approaches to Improve Interpretability

There are efforts to make RL models more transparent, making them more acceptable and trustworthy in clinical practice. SHAP (Shapley Additive Explanations) and LIME (Local Interpretable Model-Agnostic Explanations) are two explainable AI (XAI) tools that can be used as an approach. These tools also allow us to explain to humans how a particular action is affected by specific features, often through visualizations. To provide insights into the decision logic of models, XAI methods can be adapted for use in RL systems [40]. Surrogate models are another type and operate by approximating the absolute decision rule(s), but in a transparent way. These models often simplify the complex decision-making process by penalizing actions such as being "hit" in a game, so physicians can understand how the reasoning works [41]. Improvement in interpretability also happens with feature attribution methods. These methods identify and rank the importance of the input features that affect action selection. To understand which features most significantly impact the model’s decision in the case of non-linear, multi-layer RL models, techniques such as integrated gradients or layer-wise relevance propagation can be used [42].

Clinical Decision Support Systems

**Figure 2:**
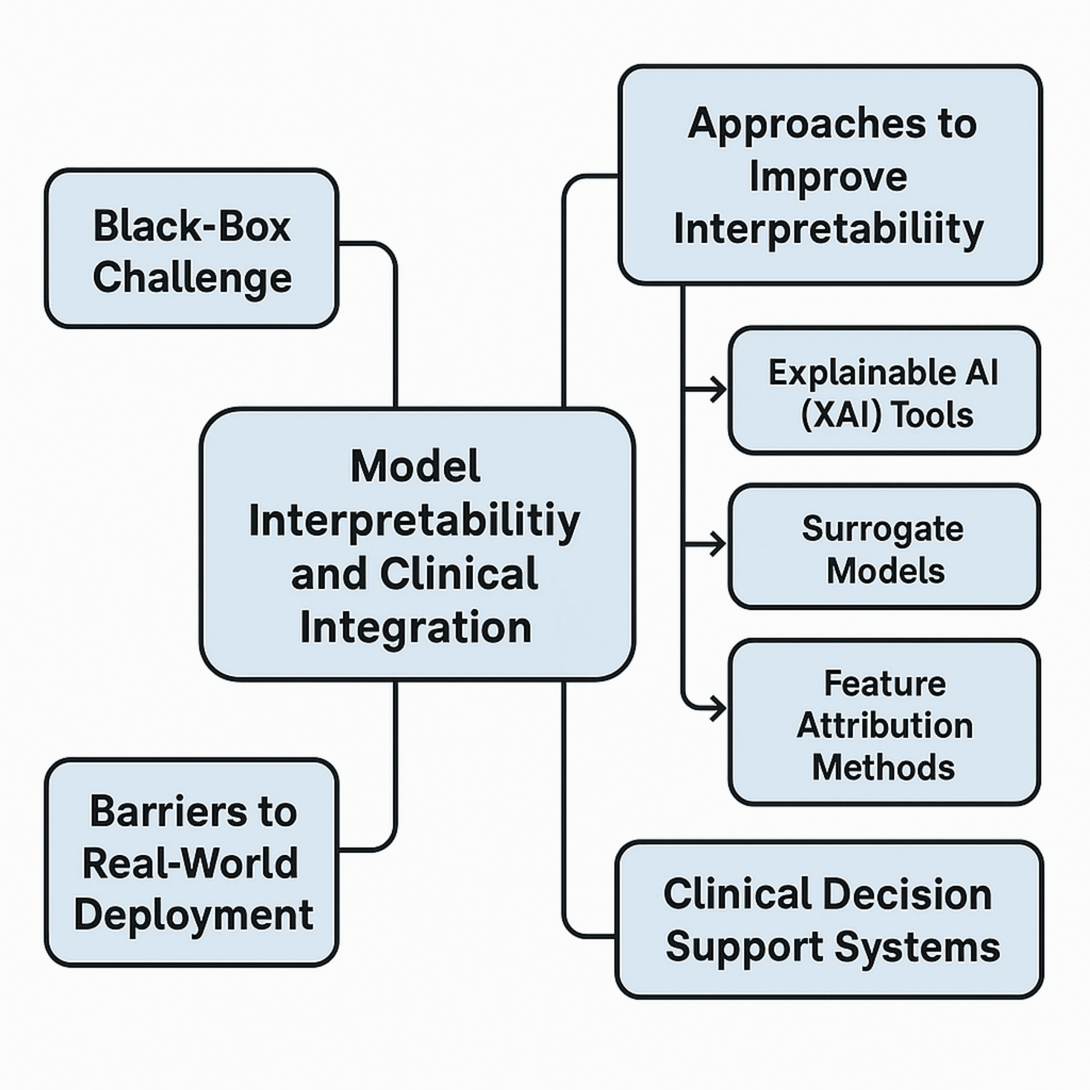
Model interpretability and clinical integration in RL for personalized medicine Image Credit: Banumathi K (Author) RL: reinforcement learning, AI: artificial intelligence, XAI: two explainable AI

RL models have to be embedded in familiar clinical workflows and not replace clinical judgment to have an impact in clinical settings. Clinical decision support systems are used to integrate this. Real-time decision support can be given directly at the point of care by embedding these RL recommendations into EHR systems. For example, they include patient-specific adaptive dosing tools for insulin and ventilator management algorithms that suggest patient-specific management decisions, which clinicians can act on for treatment [43]. Moreover, human-in-the-loop RL approaches that use clinicians in the feedback loop (e.g., by specifying the reward function or validating actions) further enhance trust, safety, and usability. To this end, using RL systems as a collaborative approach ensures that they improve clinical decision-making rather than making autonomous decisions, leading to a safer adoption of AI in healthcare [44]. A conceptual mind map of the key components required for RL to be both model interpretable and clinically integrated in a healthcare setting is presented in Figure 2.

Barriers to Real-World Deployment

While some domains are technically mature, RL models are still little deployed in real-world healthcare settings for several reasons. Retrospective dataset models are likely to fail to generalize to new environments due to changes in patient demographics, clinical practices, and data collection methods. In addition, RL algorithms demand a large amount of computational resources and infrastructure, which are not accessible in all clinical environments [45]. The expected benefits of RL depend on the successful integration of RL within hospital IT systems, which allows clinicians full access to information while maintaining hospital standards for data confidentiality, and also provides proper training for clinicians. Additionally, system updates, monitoring, and auditing must be in place to keep the model's performance over time. There is a significant barrier to clinician skepticism about AI-driven decision-making. If an RL agent's recommendation is not interpretable or is not consistently validated, clinicians may be reluctant to rely on RL to make treatment recommendations for their patients. Thus, successful deployment hinges not only on technical performance but also on usability, transparency, and alignment with clinician expectations [46].

Ethical, legal, and regulatory considerations

Patient Consent and Data Privacy

Large-scale patient data, such as EHRs, genomics, imaging, and behavioral data, are heavily relied upon by RL systems. The dependence creates major privacy issues and requires informed consent. Patients must know exactly how their data will be used in model development, as well as any secondary purposes (e.g., algorithm training or performance monitoring) [47]. To minimize the risks associated with reidentification and unauthorized access, data anonymization, differential privacy, and federated learning are being considered for achieving these objectives [47]. Still, these solutions pose challenges to scaling since healthcare institutions have varying technical capabilities and different legal requirements regarding data privacy by jurisdiction.

Algorithmic Bias and Fairness

In the absence of careful design of RL models, it is crucial to recognize that AI systems, including RL models, can perpetuate and even exacerbate existing healthcare disparities. Imbalance in training data, a non-representative patient population, or a reward structure with bias (such as noisy or corrupted data collection) can also cause biases [25]. To address these issues, it is essential to use diverse and representative datasets when training the model, which helps prevent bias. For example, RL models trained on mostly urban and insured populations may not provide good suggestions for rural or underserved populations [35]. Fairness-aware RL seeks to achieve fairness without sacrificing model performance, i.e., without harming model performance, the model should treat all populations equitably. Multi-objective optimization can be used to accomplish this by balancing clinical efficacy with fairness considerations [21].

Regulatory Guidelines for Artificial Intelligence in Healthcare

Although the AI regulatory landscape in healthcare is still emerging, the Institute of Medicine recognizes that it is a matter of great importance to address as soon as possible. The FDA and EMA are among international bodies that are also developing standards for adaptive learning systems, as these platforms present unique challenges for the use of AI in healthcare [43]. The FDA, EMA, and international standards include RL models and provisions for software as a medical device (SaMD) that permit continuously learning systems, such as RL, within the FDA SaMD framework. But the discussion is still active regarding the adaptation of these regulations to real-time learning and model updates [7]. Regimes for approving AI-driven treatment algorithms mandate that algorithms are validated and generalized to the point of being safe and effective. These models need to be validated through prospective clinical trials, post-deployment surveillance, and real-world performance audits. Finally, it is also essential to have model version control, as well as continuous performance tracking, to avoid model drift and ensure that the models remain clinically useful over time [45].

Future directions and research opportunities

Advancing Reinforcement Learning Methodologies for Healthcare

RL environments are usually more complex than healthcare environments. Hence, there is a need for methodological innovations in adapting RL techniques for clinical applications. To assist such models to adjust faster to a new task (meta-RL), handle complex clinical workflows (hierarchical RL), and coordinate care among multiple agents (e.g., doctors, nurses, or AI systems) (multi-agent RL), advanced RL architectures, namely meta-RL, hierarchical RL, and multi-agent RL, are explored [13]. Safety constraints in RL models are another essential part to consider. When working on critical tasks, such as in high-risk areas like critical care or oncology, even minor errors can have significant consequences. Safety constraints are embedded into RL models to ensure that decisions are safe and prevent harmful outcomes [24]. Constrained RL remains an active area of research, particularly in ensuring that clinical guidelines are met under limited constraints for personalized decision-making, which is crucial for healthcare applications [38].

Digital Twins and Simulated Environments

A trend is omnipresent to utilize digital twins, i.e., virtual models of patients developed from current and historical data to train and test RL. RL algorithms can explore and learn without exposing them to real patients in simulated environments. As a result, their use leads to faster learning in rare conditions or complex decision pathways that have limited real-world data [28]. Synthetic data and validation are essential in validating RL models through thought-controlled scenarios where the models can be tested and refined before being deployed in the real world. This also helps improve the model's robustness and identify potential failure points [20]. Additionally, digital twins are a valuable tool for clinical and research personnel to experiment with treatment hypotheses and observe patient outcomes over extended periods under different RL policies, helping with decision-making and optimizing care strategies [38].

Federated Learning and Privacy-Preserving Reinforcement Learning

RL model training on decentralized institutions is possible without exchanging sensitive patient data, thus ensuring data privacy. In addition to protecting patient privacy, this method increases the diversity of the training data, which may in turn improve the generalizability of the models [42]. It encourages a collaborative model of development, involving hospitals, research centers, and countries, to jointly develop personalized medicine tools worldwide, resulting in more diverse and robust outcomes for the entire healthcare system.

Interdisciplinary Collaboration

While we have described the technical challenge of applying RL to healthcare, these challenges will require cross-disciplinary collaboration. It covers AI researchers, medical professionals, bioethicists, and patients, as all of these perspectives play a role in formulating systems that are technically sound and ethically responsible. RL systems will be more clinically relevant and ethically aligned with healthcare needs by engaging in such collaborative efforts [18]. Additionally, training for clinician engagement is required to have clinicians understand and interpret RL tools. The trust could be established by offering courses, workshops, and certification programs in AI literacy for healthcare providers, enabling them to collaborate effectively with AI. This, in turn, would lead to improved human-AI collaboration and ultimately enhance patient care and outcomes [19].

Limitations and Future Research Directions

The pharmaceutical field faces substantial barriers in implementing RL for personalized treatment. The development of reward functions that translate to meaningful clinical outcomes proves to be a complex task due to the prolonged, multi-causal nature of the results. Healthcare data contains many dimensions, which causes a state space explosion that results in inefficient learning and restricted generalization [10,15]. The evaluation measures in RL remain undefined, which makes results less reproducible and limits the capacity to compare studies.

Most existing medical applications rely on historical patient data combined with simulated data, rather than conducting valid studies using real-world clinical patients [31-34]. Model fairness, along with its application scope, becomes limited due to both data quality problems and sample selection inconsistencies.

The field requires research that develops sophisticated reward engineering mechanisms, as well as studies into hierarchical learning systems, multi-agent systems, and privacy-secured approaches based on federated learning. Standardizing benchmarking tests and evaluating safe digital twin model systems will be necessary for the effective deployment of systems. To achieve safe and effective clinical care with RL, it is essential to establish both interdisciplinary collaboration and ethical oversight.

## Conclusions

RL is emerging as a transformative tool in the pursuit of personalized medicine, offering dynamic, data-driven strategies for optimizing individualized treatment protocols. Unlike traditional ML, RL's ability to continuously learn from patient feedback and adapt to changing clinical contexts makes it uniquely suited to address the complexities of modern healthcare. From oncology to chronic disease management, psychiatry, and infectious disease care, RL has shown promising applications in enhancing therapeutic precision, minimizing adverse effects, and improving long-term health outcomes. Its integration with multi-modal data, explainable AI tools, and clinical decision support systems further underscores its value in delivering context-sensitive recommendations aligned with patient-specific goals.

Despite these advances, significant challenges remain. Data privacy, algorithmic bias, model interpretability, and regulatory hurdles must be addressed to enable safe, ethical, and equitable deployment of RL systems. Future directions should prioritize interdisciplinary collaboration, clinician education, the development of digital twins, and robust validation through federated learning environments. As healthcare moves toward greater personalization, RL stands as a vital enabler, but its success will depend on responsible design, transparent integration, and inclusive governance. The time is ripe for stakeholders across medicine, data science, and policy to come together and scale RL for impactful, human-centered healthcare.
